# Aqueous Extract of Glucoraphanin-Rich Broccoli Sprouts Inhibits Formation of Advanced Glycation End Products and Attenuates Inflammatory Reactions in Endothelial Cells

**DOI:** 10.1155/2018/9823141

**Published:** 2018-08-08

**Authors:** Ami Sotokawauchi, Yuji Ishibashi, Takanori Matsui, Sho-ichi Yamagishi

**Affiliations:** Department of Pathophysiology and Therapeutics of Diabetic Vascular Complications, Kurume University School of Medicine, Kurume, Japan

## Abstract

We have previously shown that sulforaphane not only inhibits formation of advanced glycation end products (AGEs) but also exerts anti-inflammatory effects on AGE-exposed human umbilical vein endothelial cells (HUVECs) and AGE-injected rat aortae. Here we examined the effects of aqueous extract of glucoraphanin-rich broccoli sprouts on formation of AGEs and then investigated whether the extract could attenuate inflammatory or oxidative stress reactions in tumor necrosis factor-alpha (TNF-*α*)- or AGE-exposed HUVECs. Fresh broccoli sprouts were homogenized in phosphate-buffered saline and filtered through a gauze. After centrifugation, clear extract was obtained. AGE formation was measured by enzyme-linked immunosorbent assay. Gene expression was evaluated by real-time reverse transcription-polymerase chain reaction. Reactive oxygen species (ROS) generation were measured using a fluorescent dye. Five percent broccoli sprout extract inhibited the formation of AGEs, reduced basal gene expressions of monocyte chemoattractant protein-1 (MCP-1), intercellular adhesion molecule-1 (ICAM-1,) and receptor for AGEs (RAGE), and upregulated endothelial nitric oxide synthase (eNOS) mRNA levels in HUVECs. TNF-*α* upregulated MCP-1, ICAM-1, and RAGE mRNA levels in HUVECs, all of which were attenuated by the treatment with 1% broccoli sprout extract. Pretreatment of 1% broccoli sprout extract prevented the ROS generation in HUVECs evoked by AGEs. The present study demonstrates that sulforaphane-rich broccoli sprout extract could inhibit the AGE-RAGE axis and exhibit anti-inflammatory actions in HUVECs. Supplementation of sulforaphane-rich broccoli sprout extract may play a protective role against vascular injury.

## 1. Introduction

Sugars, such as glucose, glyceraldehyde, and methylglyoxal, can react nonenzymatically with the amino groups of lipids, proteins, and nucleic acids to form senescent macromolecules termed “advanced glycation end products (AGEs),” whose process has been shown to progress under inflammatory and hyperglycemic conditions [1–9]. Accumulating evidence has suggested that AGEs not only alter the structural integrity and function of macromolecules but also evoke oxidative stress generation and inflammatory reactions in various types of cells and organs through the interaction with a receptor for AGEs (RAGE), thus contributing to the development and progression of numerous aging- and diabetes-related complications, including atherosclerotic cardiovascular disease, cancer growth and metastasis, osteoporosis, and Alzheimer's disease [1–9]. These observations suggest that AGE-RAGE axis may be a novel therapeutic target of these devastating disorders.

We, along with others, have previously shown that sulforaphane inhibits the formation of AGEs* in vitro* and attenuates inflammatory reactions in AGE-exposed endothelial cells and AGE-infused rat aortae partly by reducing the expression of RAGE [10–12]. Furthermore, oral intake of sulforaphane-rich broccoli sprout extracts has been shown to exhibit chemopreventive activity against carcinogen-induced skin, breast, and stomach cancers and attenuate allergic response to air pollutants,* Helicobacter pylori*-induced gastritis, ultraviolet radiation-evoked skin damage, and fatty liver injury in humans through its anti-inflammatory properties [13–17]. However, it remains unclear whether broccoli sprout extract could exhibit the inhibitory effects on AGE-RAGE axis in endothelial cells. So, in this study, we examined the effects of aqueous extract of a precursor of sulforaphane, glucoraphanin-rich broccoli sprouts on AGE formation* in vitro* and then investigated whether the extract could attenuate the inflammatory reactions in tumor necrosis factor-*α* (TNF-*α*)- or AGE-exposed human umbilical vein endothelial cells (HUVECs).

## 2. Materials and Methods

### 2.1. Materials

Bovine serum albumin (BSA) (essentially fatty acid free) and D-glyceraldehyde were purchased from Sigma (St. Louis, MO, USA). TNF-*α* was purchased from Cell Signaling Technology (Danvers, MA, USA).

### 2.2. Preparation of AGE-BSA

AGE-BSA was prepared as described previously [10]. In brief, BSA (25 mg/ml) was incubated under sterile conditions with 0.1 M glyceraldehyde in 0.2 M NaPO_4_ buffer (pH 7.4) at 37°C for 7 days. Then unincorporated sugars were removed by dialysis with phosphate-buffered saline. Control nonglycated BSA was incubated in the same conditions except for the absence of D-glyceraldehyde as described previously [10].

### 2.3. Preparation of Sulforaphane-Rich Broccoli Sprout Extract

Glucoraphanin-rich broccoli sprouts containing* ca.* 6 *μ*mol glucoraphanin/g broccoli sprouts were identified by Fahey et al. [18] and cultured at Murakami Farm, Hiroshima, Japan, where voucher specimens were deposited (Broccoli Super Sprout, Part No. 3359642). Glucoraphanin-rich broccoli sprouts were obtained from a local supermarket. Fresh broccoli sprouts (50 g) were suspended and homogenized in 50 ml phosphate-buffered saline (pH 7.4) with a blender and filtered through a gauze. After centrifugation at 3,000 g for 30 minutes at 4°C, clear extract was obtained and stored at -30°C. Broccoli sprout extract was diluted with phosphate-buffered saline or cell culture medium to make final concentrations of 1% and 5% broccoli sprout extract.

### 2.4. Measurement of AGEs

BSA (25 mg/ml) was incubated with 1 mM glyceraldehyde in the presence or absence of the indicated concentrations of broccoli sprout extract for 1 day, and then levels of AGEs were measured with enzyme-linked immunosorbent assay as described previously [19]. Intra- and interassay coefficients of variation of the assay were 6 and 2.6%, respectively.

### 2.5. Cells

HUVECs obtained from Lonza Group Ltd. (Basel, Switzerland) were cultured in endothelial basal medium supplemented with 2% fetal bovine serum, 0.4% bovine brain extracts, 10 ng/ml human epidermal growth factor, and 1 *μ*g/ml hydrocortisone according to the manufacturer's recommendation. Treatments with TNF-*α*, AGEs, and/or broccoli sprout extract were carried out in a medium lacking epidermal growth factor and hydrocortisone.

### 2.6. Real-Time Reverse Transcription-Polymerase Chain Reactions (RT-PCR)

HUVECs were treated with or without 10 ng/ml TNF-*α* in the presence or absence of the indicated concentrations of broccoli sprout extract for 4 hours. Then total RNA was extracted with RNAqueous-4PCR kit (Ambion Inc., Austin, TX, USA) according to the supplier's instructions. Quantitative real-time RT-PCR was performed using Assay-on-Demand and TaqMan 5 fluorogenic nuclease chemistry (Applied Biosystems, Foster city, CA, USA) according to the manufacturer's recommendation. IDs of primers for human monocyte chemoattractant protein-1 (MCP-1), intercellular adhesion molecule-1 (ICAM-1), RAGE, endothelial nitric oxide synthase (eNOS), *β*-actin, and 18S rRNA gene were Hs00234140_m1, Hs00164932_m1, Hs00542592_g1, Hs01574659_m1, Hs99999903_m1, and Hs99999901_s1, respectively.

### 2.7. Measurement of Reactive Oxygen Species (ROS) Production

ROS generation was measured by using a fluorescent probe, carboxy-H_2_DFFDA at (Thermo Fisher Scientific, Waltham, MA, USA) as described previously [10]. In brief, HUVECs were treated with or without 1% broccoli sprout extract for 4 hours. After washing the cells with phosphate-buffered saline, cells were incubated with 100 *μ*g/ml AGEs or 100 *μ*g/ml nonglycated BSA in the presence of 1 *μ*M carboxy-H_2_DFFDA for 25 minutes, and then fluorescence intensity was measured.

### 2.8. Statistical Analysis

**Figure 1:**
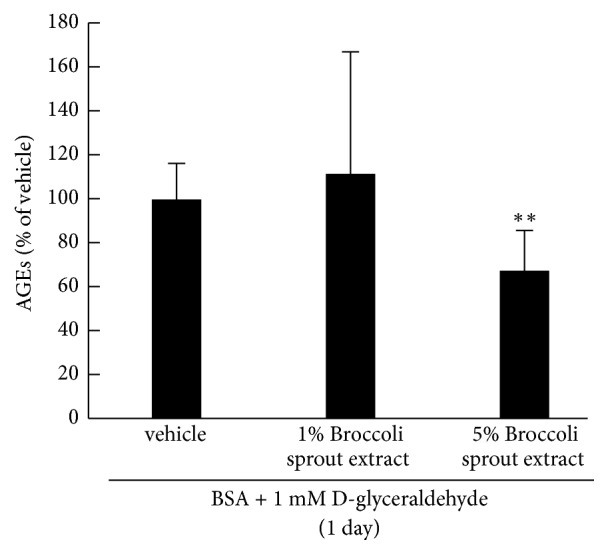
**Effects of broccoli sprout extract on formation of AGEs* in vitro***. BSA was incubated with 1 mM glyceraldehyde in the presence or absence of the indicated concentrations of glucoraphanin-rich broccoli sprout extract for 1 day, and then levels of AGEs were measured with enzyme-linked immunosorbent assay.* N* = 3 per group. *∗∗*, p < 0.01 compared to the control values with vehicle.

**Figure 2:**
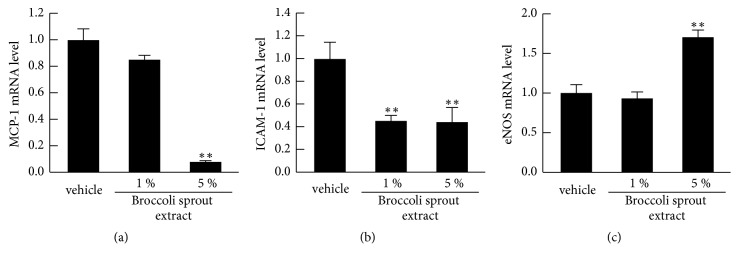
**Effects of broccoli sprout extract on MCP-1 (a), ICAM-1 (b), and eNOS mRNA levels (c) in HUVECs**. HUVECs were treated with or without the indicated concentrations of glucoraphanin-rich broccoli sprout extract for 4 hours. Then total RNAs were transcribed and amplified by real-time PCR. Data were normalized by the intensity of *β*-actin mRNA- (a and b) or 18S rRNA-derived signals (c) and then related to the control values with vehicle.* N* = 3 per group. *∗∗*, p < 0.01 compared to the control values with vehicle.

**Figure 3:**
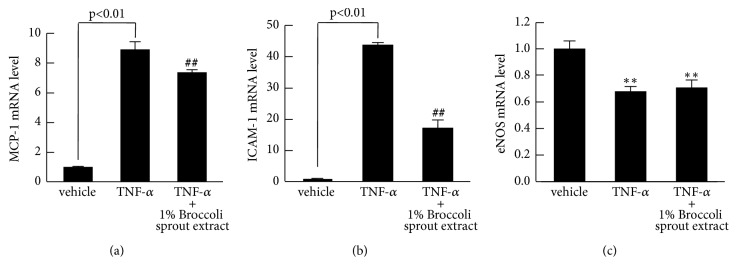
**Effects of broccoli sprout extract on MCP-1 (a), ICAM-1 (b), and eNOS mRNA levels (c) in TNF-**α**-exposed HUVECs**. HUVECs were treated with or without 10 ng/ml TNF-*α* in the presence or absence of 1% of glucoraphanin-rich broccoli sprout extract for 4 hours. Then total RNAs were transcribed and amplified by real-time PCR. Data were normalized by the intensity of 18S rRNA-derived signals and then related to the values with vehicle.* N* = 3 per group. ##, p<0.01 compared to the values with TNF-*α* alone. *∗∗*, p < 0.01 compared to the control values with vehicle.

**Figure 4:**
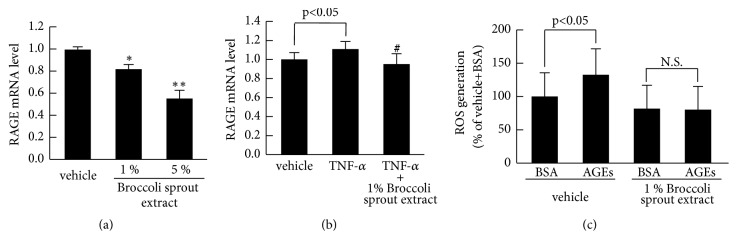
**Effects of broccoli sprout extract on RAGE mRNA levels (a and b) and ROS generation (c) in HUVECs**. (a and b) HUVECs were treated with or without 10 ng/ml TNF-*α* in the presence or absence of 1% of glucoraphanin-rich broccoli sprout extract for 4 hours. Then total RNAs were transcribed and amplified by real-time PCR. Data were normalized by the intensity of *β*-actin mRNA- (a) or 18S rRNA-derived signals (b) and then related to the values with vehicle. (c) HUVECs were treated with or without 1% broccoli sprout extract for 4 hours. After washing the cells with phosphate-buffered saline, cells were incubated with 100 *μ*g/ml AGEs or 100 *μ*g/ml nonglycated BSA in the presence of carboxy-H_2_DFFDA for 25 minutes, and then fluorescence intensity was measured. *∗* and *∗∗*, p < 0.05 and p < 0.01 compared to the control values with vehicle, respectively. #, p < 0.05 compared to the values with TNF-*α* alone. N.S: not significant.

All values were presented as mean ± standard deviation. One-way ANOVA followed by Tukey's HSD test for Figures 2(b), 2(c), and 4(a), Games-Howell test for Figures 1 and 2(a), Steel-Dwass test for Figure 4(c), and Student's* t*-test for Figures 3 and 4(b) was performed for statistical comparisons; p < 0.05 was considered significant.

## 3. Results

We first examined the effects of broccoli sprout extract on formation of AGEs. As shown in Figure 1, Incubation of BSA with 1 mM glyceraldehyde for 1 day increased the formation of AGEs, which was significantly inhibited by 5%, but not 1% broccoli sprout extract.

A four-hour incubation of 5% of broccoli sprout extract significantly decreased basal MCP-1 and ICAM-1 mRNA levels in HUVECs, while it upregulated eNOS mRNA levels (Figure 2). However, 1% of broccoli sprout extract did not affect these gene expressions except for ICAM-1; basal ICAM-1 mRNA levels were decreased to 40% of control cells by the treatment with 1% broccoli sprout extract (Figure 2(b)).

We next investigated the effects of 1% broccoli sprout extract on MCP-1, ICAM-1, and eNOS gene expressions in TNF-*α*-exposed HUVECs. As shown in Figures 3(a) and 3(b), TNF-*α* upregulated mRNA levels of MCP-1 and ICAM-1, which were significantly inhibited by 1% broccoli sprout extract. TNF-*α* reduced eNOS gene expression in HUVECs, but it was not affected by 1% broccoli sprout extract (Figure 3(c)).

We further examined the effects of broccoli sprout extract on RAGE gene expression in HUVECs. As shown in Figure 4(a), broccoli sprout extract dose-dependently reduced basal RAGE mRNA levels in HUVECs. Moreover, 1% broccoli sprout extract significantly inhibited the TNF-*α*-induced upregulation of RAGE mRNA levels in HUVECs (Figure 4(b)). Although AGEs elicited ROS generation in vehicle-pretreated HUVECs, the ROS-inducing effect of AGEs was not observed in 1% broccoli sprout extract-pretreated HUVECs (Figure 4(c)).

## 4. Discussion

In this study, we found for the first time that 5%, but not 1% sulforaphane-rich broccoli sprout extract, significantly inhibited the formation of AGEs* in vitro*. When broccoli sprouts are extracted in boiling water and the extract is treated with active daikon myrosinase, about half of glucoraphanin, a precursor of sulforaphane in the broccoli sprouts has been shown to be converted to sulforaphane [20]. So, if we assume that extraction efficiency of cold water is about 1/3~2/3 of that of boiling water, since glucoraphanin in fresh broccoli sprouts is hydrolyzed by endogenous myrosinase and converted to sulforaphane with about 50% efficiency [18], 1% and 5% broccoli sprout extracts used for the present experiments are estimated to contain* ca.* 2.5~5 and 12.5~25 *μ*M sulforaphane, respectively. Based on our previous finding that 25 *μ*M, but not 0.4 *μ*M sulforaphane, inhibited the formation of AGEs* in vitro* [12], the inhibitory effects of 5% broccoli sprout extract on AGE formation could be attributed largely to its high content of sulforaphane. Daily intake of 25 g glucoraphanin-rich broccoli sprouts for 2 months significantly decreased serum levels of AGEs in healthy volunteers by 20%, which were accompanied with the decreased ratio of AGEs to soluble form of RAGE, a marker of the activation of AGE-RAGE axis [12, 21]. Taken together, these observations suggest that consumption of sulforaphane-rich broccoli sprout extract may attenuate the activation of AGE-RAGE system in human body.

We found here that 5% broccoli sprout extract for 4 hours decreased basal gene expression levels of MCP-1, ICAM-1, and RAGE in HUVECs by 50-90%, while it increased eNOS mRNA levels to 1.5-fold over the control values. So, it is unlikely that broccoli sprout extract at this concentration may exert toxic effects on HUVECs. However, since the effects of 5% broccoli sprout extract on nontreated HUVECs were drastic, we chose the concentration of 1% broccoli sprout extract in the experiments of TNF-*α* - or AGE-exposed HUVECs. In the present study, we also found that TNF-*α*-induced upregulation of MCP-1, ICAM-1, and RAGE mRNA levels and was significantly attenuated by 1% broccoli sprout extract. The extent of decreases in MCP-1, ICAM-1, and RAGE mRNA levels by 1% broccoli sprout extract was almost similar in both nontreated and TNF-*α*-exposed HUVECs. Sulforaphane at 2.5 *μ*M has been shown to stimulate nuclear translocation of NF-E2-related factor-2 (Nrf2) and induce phase II anti-oxidative enzymes, thus resulting in suppression of a redox-sensitive transcriptional factor, nuclear factor-*κ*B (NF-*κ*B) activation [22]. Given that MCP-1, ICAM-1, and RAGE gene expressions are transcriptionally regulated by NF-*κ*B [10, 23], 1% broccoli sprout extract could reduce basal and TNF-*α*-induced MCP-1, ICAM-1, and RAGE mRNA levels in HUVECs via inhibition of NF-*κ*B activation, which may be dependent on sulforaphane-induced Nrf2 activation. In support of our speculation, 5 *μ*M sulforaphane, which is assumed to be contained in 1% broccoli sprout extract, has been reported to inhibit oxidazed low-density lipoprotein-induced oxidative stress generation and inflammatory reactions in HUVECs, whose beneficial effects are attenuated by Nrf2 knockdown [24]. Furthermore, we have previously shown that 1.6 *μ*M sulforaphane suppresses oxidative stress generation and inflammatory reactions in AGE-exposed HUVECs as well [10]. These findings suggest that as is the case for AGE formation, anti-inflammatory effects of broccoli sprout extract in HUVECs could be largely ascribed to sulforaphane. However, we cannot totally exclude the possibility that other phytochemicals contained in broccoli sprout extract may exert anti-inflammatory effects. Indeed, *β*-carotene in combination with quercetin or vitamin C and E is reported to restore antioxidant enzyme activities and inhibit NF-*κ*B activation in the lungs of benzo[a]pyren-exposed Mongolian gerbils [25]. Moreover, quercetin and sulforaphane act additively to inhibit ROS generation and MCP-1 and RAGE gene expression in AGE-exposed HUVECs [12]. Although quercetin is one of the main flavonoids contained in broccoli sprout (50 *μ*g quercetin/g broccoli sprout) [26], it is unlikely that quercetin could work as an anti-inflammatory agent in our present study because it is practically water insoluble [26, 27].

We, along with others, have previously shown that engagement of RAGE with AGEs evokes oxidative stress generation and inflammatory reactions in HUVECs [28–30]. In this study, 1% broccoli sprout extract not only reduced RAGE mRNA levels in both nontreated and TNF-*α*-treated HUVECs but also blocked the ROS generation elicited by AGEs. Therefore, broccoli sprout extract may attenuate the activation of AGE-RAGE pathway by at least two mechanisms: one is the inhibition of AGE formation and the other is the suppression of RAGE expression. Since the AGE-RAGE interaction-mediated ROS generation could further stimulate the formation of AGEs and induction of RAGE [31–33], broccoli sprout extract may break the vicious cycle between the AGE-RAGE axis and ROS production.

Accumulating evidence has suggested that MCP-1 and ICAM-1 stimulate the recruitment and firm adhesion of inflammatory cells to endothelial cells, thereby contributing to the develop and progression of atherosclerosis [34–36]. Furthermore, endothelial cell-derived nitric oxide not only protects against endothelial dysfunction but also inhibits inflammatory and thrombotic reactions, thus playing a crucial role in the maintenance of vascular hemeostasis [37, 38]. In addition, impaired endothelial function is associated with reduced activity of Nrf2 in the arterial tissue of rats with chronic kidney disease [39], while activation of Nrf2 has been shown to increase eNOS expression and phosphorylation in endothelial cells [40]. These observations suggest that broccoli sprout extract may play a protective role against vascular injury through its anti-inflammatory properties partly via activation of Nrf2-nitric oxide system.

## 5. Conclusions

In this study, we could not clarify active components in broccoli sprout extract that might be responsible for the observed effects. However, the aim of the present study is to clarify whether aqueous extract of sulforaphane-rich broccoli sprout could inhibit the AGE-RAGE axis and exhibit anti-inflammatory effects in HUVECs as in the case of sulforaphane [10, 12]. Our present study suggests that glucoraphanin-rich broccoli sprout extract may exert beneficial actions against vascular injury via several mechanisms, such as the inhibition of AGE formation, suppression of inflammatory reactions, reduction of RAGE expression, and upregulation of eNOS mRNA levels. Further longitudinal clinical study is needed to clarify whether supplementation of sulforaphane-rich broccoli sprout extract could prevent the development and progression of atherosclerotic cardiovascular disease.

## Data Availability

The datasets used and/or analyzed during the current study available from the corresponding author on reasonable request.
